# Integrated Transcriptome and Metabolome Analysis Reveals Key Metabolites Involved in *Camellia oleifera* Defense against Anthracnose

**DOI:** 10.3390/ijms23010536

**Published:** 2022-01-04

**Authors:** Chaochen Yang, Pengfei Wu, Xiaohua Yao, Yu Sheng, Chengcai Zhang, Ping Lin, Kailiang Wang

**Affiliations:** Research Institute of Subtropical Forestry, Chinese Academy of Forestry, Hangzhou 311400, China; ylsdr2019jjl@163.com (C.Y.); 15570894428@163.com (P.W.); shengyu2000@foxmail.com (Y.S.); c.c.zhang@caf.ac.cn (C.Z.); linping80@126.com (P.L.); wangkl163@163.com (K.W.)

**Keywords:** *Camellia oleifera*, anthracnose, *Colletotrichum gloeosporioides*, transcriptome, metabolome, flavonoid biosynthesis

## Abstract

*Camellia oleifera* (*Ca. oleifera*) is a woody tree species cultivated for the production of edible oil from its seed. The growth and yield of tea-oil trees are severely affected by anthracnose (caused by *Colletotrichum gloeosporioides*). In this study, the transcriptomic and metabolomic analyses were performed to detect the key transcripts and metabolites associated with differences in the susceptibility between anthracnose-resistant (ChangLin150) and susceptible (ChangLin102) varieties of *Ca. oleifera*. In total, 5001 differentially expressed genes (DEGs) were obtained, of which 479 DEGs were common between the susceptible and resistant varieties and further analyzed. KEGG enrichment analysis showed that these DEGs were significantly enriched in tyrosine metabolism, phenylpropanoid biosynthesis, flavonoid biosynthesis and isoquinoline alkaloid biosynthesis pathways. Furthermore, 68 differentially accumulated metabolites (DAMs) were detected, including flavonoids, such as epicatechin, phenethyl caffeate and procyanidin B2. Comparison of the DEGs and DAMs revealed that epicatechin, procyanidin B2 and arachidonic acid (peroxide free) are potentially important. The expression patterns of genes involved in flavonoid biosynthesis were confirmed by qRT-PCR. These results suggested that flavonoid biosynthesis might play an important role in the fight against anthracnose. This study provides valuable molecular information about the response of *Ca. oleifera* to *Co. gloeosporioides* infection and will aid the selection of resistant varieties using marker-assisted breeding.

## 1. Introduction

*Camellia oleifera* (*Ca. oleifera*) is a commercial shrub native to China that has been widely grown in southern China for over 2000 years [1,2,3]. *Ca. oleifera* is one of four woody edible oil crops in China [4]. The tea oil extracted from its seed, similar to olive oil, is an edible oil and is popular in Chinese cooking [5,6,7]. *Ca. oleifera* are notable not only as an important source of edible oil but also for environmental protection by limiting soil erosion [8,9]. However, *Ca. oleifera* is susceptible to many bacterial, fungal and viral diseases, which threaten the healthy development of the tea-oil industry. Of these diseases, anthracnose, caused by Colletotrichum, seriously affects tea-oil yield and quality. In China, *Colletotrichum gloeosporioides* (*Co. gloeosporioides*) is the predominant anthracnose pathogen affecting *Ca. oleifera* [10,11,12]. It can infect various plant organs, mainly the leaves and stalks, resulting in anthracnose stalk rot (ASR) and anthracnose leaf blight (ALB), which leads to the death of branches and sometimes entire plants [13,14].

*Co. gloeosporioides* is a hemibiotrophic parasitic fungus that enters the plant tissue via stomata, hydathodes, or wounds and proliferates in intercellular spaces [15]. The fungus can quickly spread under warm and humid conditions and is difficult to control. *Colletotrichum spp*. Cause diseases in many plants, including specialty crops like mango [16], tea [17], chili [18,19], olive [20], citrus [21]. Anthracnose causes severe economic losses and poses a huge threat to agriculture and forestry globally. Many approaches have been used to reduce the losses caused by anthracnose, including agronomic measures and host resistance, but these measures are not always feasible [22]. Chemical pesticides can prevent anthracnose, but the resistance of the pathogens develops easily [23]. Therefore, breeding and utilization of resistant varieties would be an economic and effective approach to manage diseases in long-lived species like the tea-oil tree. However, the molecular mechanisms underlying resistance to anthracnose in *Ca. oleifera* are largely unknown.

In recent years, joint analysis of transcriptome and metabolome data has revealed aspects of the response of plants to biological stressors [24,25], including the interaction of plants and pathogens. For example, flavonoid biosynthesis plays an important role in the response of the herbaceous plant Stylosanthes to anthracnose infection [26]. A chitin elicitor receptor kinase gene (*HvCERK1*) was highly expressed in disease-resistant barley varieties and may be a key gene for resistance to Fusarium head blight [27]. An integrated, de novo transcriptome and metabolome approach has revealed that cell structure and hormone levels change in common bean after infection with Fusarium [28]. The combined analysis of the transcriptome and the metabolome can contribute to our understanding of plant-pathogen interactions and the identification of resistance genes and disease resistant varieties [24]. The current research on *Ca. oleifera* infection by *Co. gloeosporioides* focuses on the isolation and identification of the pathogen, the pathogenic mechanism of the pathogen, etc. [10,14,29,30]. However, there are few studies on the interaction between *Ca. oleifera* and *Colletotrichum* that combine transcriptomics and metabolomics.

In previous research, we had isolated two *Ca. oleifera* varieties, Changlin102 (CL102, susceptible) and Changlin150 (CL150, resistant) that showed different sensitivities to *Co. gloeosporioides* infection [31]. In the current study, transcriptome and metabolome analyses were combined to reveal candidate genes and pathways that may respond to *Co. gloeosporioides* infection, with the expectation of providing a better understanding of *Ca. oleifera* defense mechanisms to *Co. gloeosporioides* infection.

## 2. Results

### 2.1. Phenotypes of Two Ca. oleifera Varieties after Infection by Co. gloeosporioides

**Figure 1:**
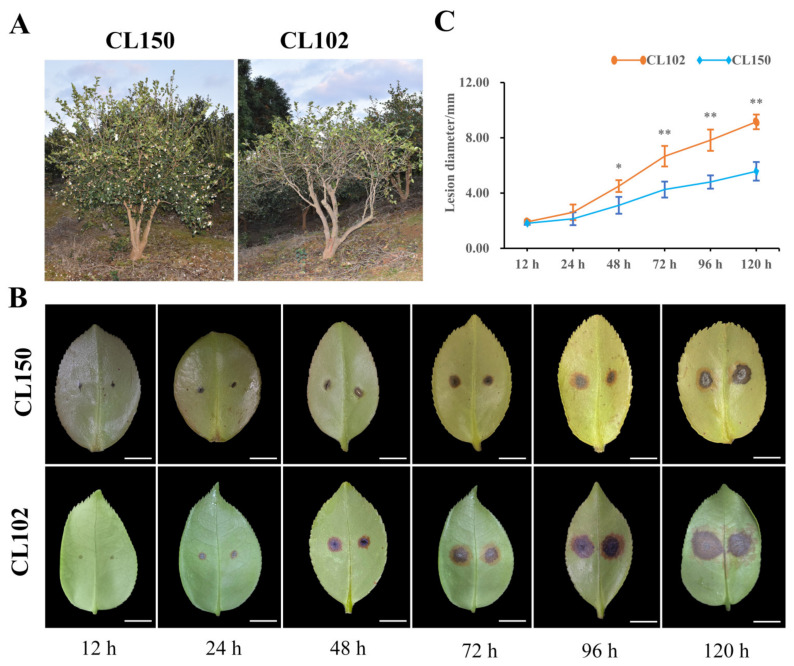
(**A**) Growth of CL150 and CL102 in the field. (**B**) Phenotype of CL150 and CL102 after inoculation with *Co. gloeosporioides*, Scale bar = 1 cm. (**C**) Leaf lesion size over time of CL150 and CL102 after inoculation with *Co. Gloeosporioides*, *n* = 12; “*” and “**” indicate significant differences at *p* < 0.05 and *p* < 0.01, respectively.

Our previous survey of cultivated accessions in the field indicated that the individuals CL150 and CL102 showed different resistance levels to anthracnose. CL150 was more resistant to anthracnose and grew significantly better than CL102 (Figure 1A). To verify the results of the field survey, *Co. gloeosporioides* was inoculated on young, wounded leaves of CL102 and CL150. Infected leaves of both lines showed obvious symptoms of anthracnose. First, brown or black spots appeared, and then the spots gradually increased (Figure 1B). The lesion diameters were measured during disease progression. At 48 h (h) after inoculation with *Co. gloeosporioides*, the lesion diameter of CL102 was significantly larger (at *p* < 0.05) than that of CL105. By 72 h, the difference was even greater (Figure 1C).

### 2.2. Differentially Expressed Genes (DEGs) Analysis

**Figure 2:**
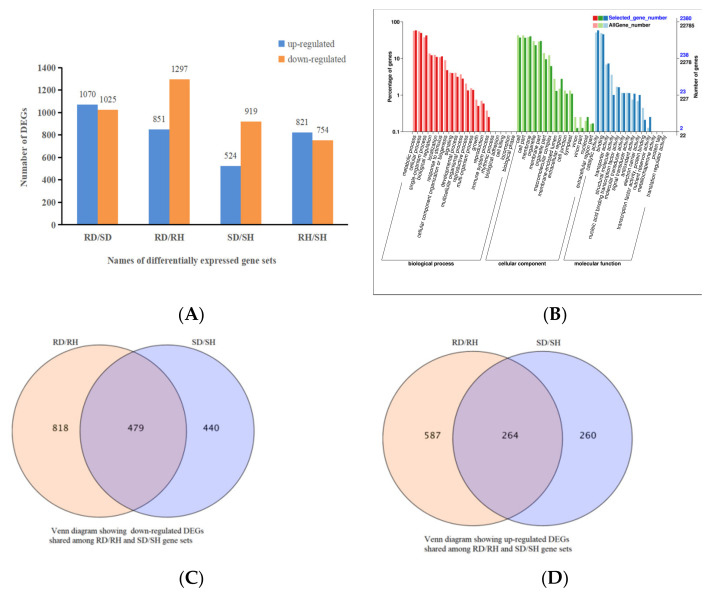
(**A**) Number of up/down-regulated DEGs between the four groups (RD/SD, RD/RH, SD/SH and RH/SH); (**B**) GO functional enrichment analysis of DEGs; (**C**) Venn diagram showing down-regulated DEGs shared among RD/RH and SD/SH; (**D**) Venn diagram showing up-regulated DEGs shared among RD/RH and SD/SH.

To analyze the transcriptional response in *Ca. oleifera* leaves to *Co. gloeosporioides* infection, young leaves were inoculated with bacterial cake (5 mm). After 72 h, healthy (uninoculated CL150 was named RH and CL102 was named SH) and diseased (inoculated CL150 was named RD and CL102 was named SD) leaves were collected for RNA-seq analysis. A total of 82.75 Gb clean data were obtained. The numbers of clean reads for each sample ranged from 19,433,566 to 37,417,417 with a Q30-value of >91.50% and GC content 44.62–45.32% (Table S1). After de novo assembling, 215,526 transcripts were generated, with a mean length of 1001.7 bp and an N50 of 1561 bp. After removing the redundant transcripts, 100,449 unigenes were obtained, with a mean length of 781.52 bp and an N50 length of 1309 bp (Table S1). In the comparison of gene expression in uninoculated and inoculated leaves of the resistant CL150, there were 2148 DEGs, with 851 up-regulated and 1297 down-regulated genes. In the susceptible line, there were 1443 DEGs uninfected and infected leaves, of which 524 DEGs were up-regulated and 919 DEGs were down-regulated. Between the infected resistant and susceptible lines, there were 2095 DEGs, of which 1070 were up-regulated and 1025 were down-regulated. Between the uninfected resistant and susceptible lines, there were 1575 DEGs, with 821 up-regulated and 754 down-regulated (Figure 2A). All DEGs, annotated in the GO database, were divided into several categories: biological process (the top three GO terms were metabolic process, cellular process and single-organism process), cellular component (cell, cell part and membrane) and molecular function (catalytic activity, binding and transporter activity) (Figure 2B). When these results were illustrated as a Venn diagram, it was clear that both unique and shared DEGs were identified between, and among, the pairs (Figure S1). By observing the DEGs throughout the infection time course, 479 DEGs were found to be down-regulated and 264 DEGs up-regulated in infected/uninfected pairwise comparisons of both genotypes (Figure 2C,D). Next, 479 down-regulated DEGs were mapped to Kyoto Encyclopedia of Genes and Genomes (KEGG) pathways. A total of 15 KEGG pathways were significantly enriched, with the most abundant being “tyrosine metabolism”, followed by “phenylpropanoid biosynthesis”, “flavonoid biosynthesis” and “isoquinoline alkaloid biosynthesis” (Table 1). Finally, there were 264 up-regulated DEGs significantly enriched in six KEGG pathways, the most abundant was “photosynthesis”, followed by “carbon fixation in photosynthetic organisms” and “thiamine metabolism” (Figure S2).

### 2.3. Co. gloeosporioides Infection Alters Metabolite Profiles in Ca. oleifera 

**Figure 3:**
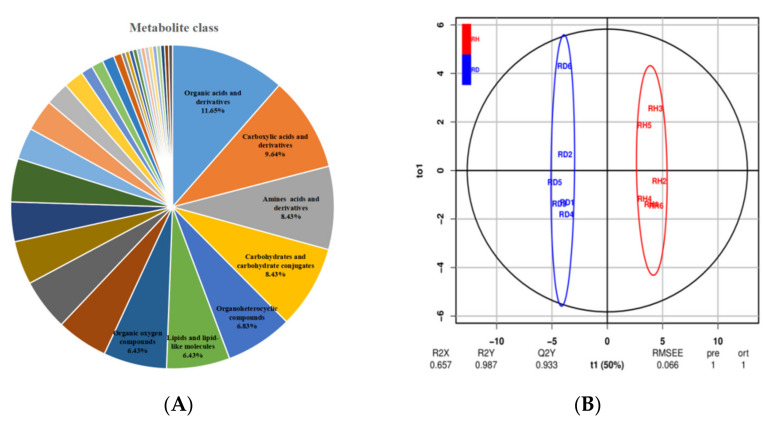
(**A**) Sector graph of metabolite classification. (**B**) OPLS-DA scatter plot of resistant line sample infected or uninfected with Co. gloeosporioides.

To gain insight into the metabolic changes that occur following *Co. gloeosporioides* infection, untargeted metabolomics by UHPLC-QTOF-MS was performed to broadly evaluate metabolite profiles in healthy and diseased leaves at 72 h. Our metabolomics data identified 249 metabolites with known structures in *Ca. oleifera*, divided into 33 categories. Among them, seven categories account for more than 6% of the metabolites, including organic acids and derivatives (11.65%), carboxylic acids and derivatives (9.64%), amino acids and derivatives (8.43%), carbohydrates and carbohydrate conjugates (8.43%), organoheterocyclic compounds (6.83%), lipids and lipid-like molecules (6.43%), and organic oxygen compounds (6.43%) (Figure 3A; Supplementary Table S2). A combined method of OPLS-DA and single-dimensional analysis (*t*-test)was used to better screen different metabolites between different groups. The OPLS-DA demonstrated a clear separation between the uninfected and infected resistant samples (Figure 3B) (Figures S3–S5), which indicated that the metabolites of uninfected and infected leaves were significantly altered.

**Figure 4:**
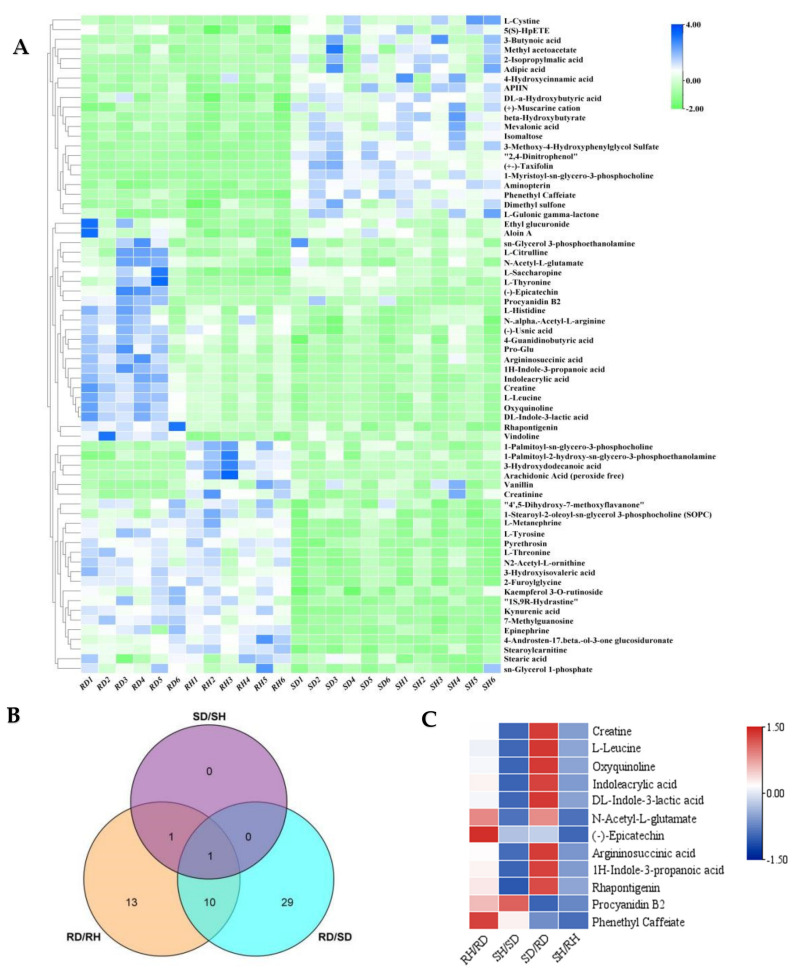
(**A**) Heat map of 68 differentially accumulated metabolites (DAMs), data of each row was standardized. (**B**) Venn diagram showing DAMs shared among RD/RH, SD/SH and RD/SD. (**C**) Heat map of fold changes of 12 DAMs in four pairwise comparsions, data of each row was standardized.

**Figure 5:**
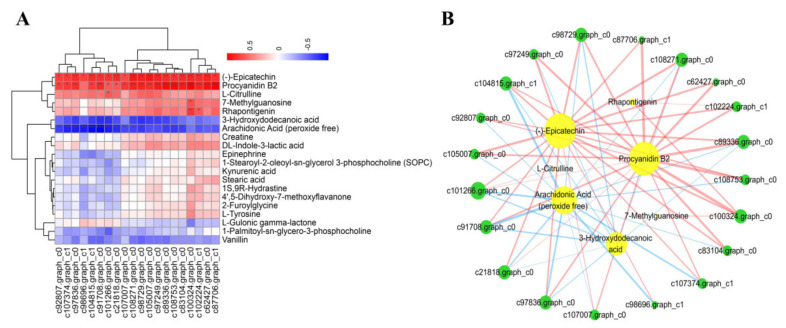
Analysis of the correlation between the changes in levels of DEGs and DAMs. (**A**) Correlation heat map. Asterisks in the panels represent the significance of the correlation, with *p*-values of less than 0.001 (***), less than 0.01 (**), and less than 0.05 (*). (**B**) Associated network diagram created using Cytoscape. Lines colored in “red” and “blue” represent positive and negative correlations, yellow circles indicate metabolites and green circles indicate genes, *p*-value set at 0.05.

There were 68 differentially accumulated metabolites (DAMs) among the four test groups, which were divided into three categories. The first type of metabolite was highly expressed in the susceptible tree line, regardless of the infection state. The second type was highly expressed in the infected resistant leaves, while the third type was highly expressed in resistant leaves regardless of the infection state (Figure 4A). There were between two and thirty-eight significant DAMs within each pairwise comparison, details of which are given in Supplementary Table S3. Among them, two DAMs were down-regulated in the infected/uninfected pairs regardless of genotype, 11 DAMs were shared by RH/RD and RD/SD, and one was shared by RH/RD, SD/SH and RD/SD (Figure 4B). The largest change in accumulation for three of the DAMs (epicatechin, phenethyl caffeate, N-Acetyl-L-glutamate) was between the infected/uninfected resistant leaves. Procyanidin B2 showed the largest change in the uninfected/infected susceptible comparison. Another eight metabolites (DL-Indole-3-lactic acid, 1S,9R-Hydrastine, Rhapontigenin, etc.) showed the greatest changes in accumulation in the comparison RH/RD (Figure 5).

### 2.4. Integrated Transcriptomic and Metabolomic Analysis to Detect Key Candidate Genes and Metabolites for Co. gloeosporioides Resistance in Ca. oleifera

The Pearson correlation algorithm was used to calculate the correlation between genes and metabolites using the relative content data from the two analyses. A correlation heat map was drawn for the top 20 results (Figure 5A). The DEGs (c92807.graph_c0, c107374.graph_c1, c97836.graph_c0, c98696.graph_c0, c104815.graph_c1, c91708.graph_c0, c101266.graph_c0, c21818.graph_c0, c107007.graph_c0, c108271.graph_c0, c98729.graph_c0, c105007.graph_c0, c97249.graph_c0, c89336.graph_c0, c108753.graph_c0, c83104.graph_c0, c1000324.graph_c0, c102224.graph_c1, c62427.graph_c0, c87706.graph_c1) were positively correlated wich —(-) epicatechin, procyanidin B2, 3-hydroxydodecanoic acid and arachidonic acid (peroxide free). These associations were confirmed by association network diagram (Figure 5B).

### 2.5. Tyrosine Metabolism in Ca. oleifera Leaves Responds to Co. gloeosporioides Infection

**Figure 6:**
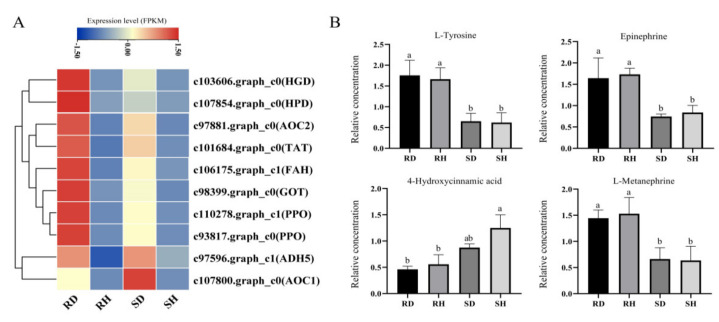
(**A**) Expression of DEGs in tyrosine metabolism pathway among the four sample groups, data of each row was standardized. (**B**) Relative concentration of four DAMs in tyrosine metabolism pathway among the four sample groups, different letters represent significant difference at *p* < 0.05 by Duncan.

Ten DEGs in the tyrosine metabolism pathway were identified (Figure 6A). Interestingly, the DEGs of both resistant and susceptible lines showed higher expression in inoculated than uninoculated leaves. Two DEGs were most highly expressed in inoculated susceptible lines and eight DEGs were most highly expressed in the inoculated resistant line. The two polyphenol oxidase (PPO) genes (c110278.graph_c1 and c93817.graph_c0) showed consistent expression trends, while the two (primary-amine oxidase) AOC genes showed different expression trends, with c97881.graph_c0 being highly expressed in inoculated resistant line and c107800.graph_c0 being highly expressed in inoculated susceptible line (Figure 6A). Among the four differential metabolites that we detected in the tyrosine metabolism pathway, L-Tyrosine, Epinephrine, and L-Metanephrine showed a consistent trend, all being significantly higher content in resistant line than in susceptible line. While 4-Hydroxycinnamic acid content was higher in the susceptible line than in the resistant line. The four metabolites contents showed no significant differences between RH/RD, SH/SD (Figure 6B).

### 2.6. Phenylpropanoid and Flavonoid Biosynthesis Induced by Co. gloeosporioides

**Figure 7:**
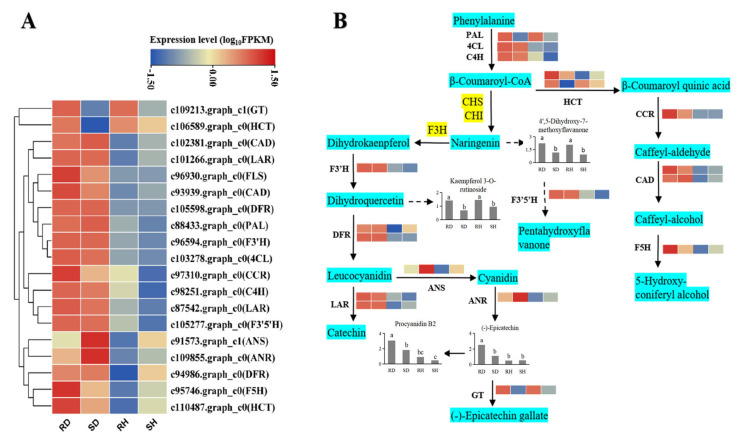
Expression of candidate genes involved in flavonoid biosynthesis. (**A**) The expression profile of key genes among the four sample groups, data of each row was standardized. (**B**) The postulated biosynthetic pathway of flavonoids after *Co. gloeosporioides* infection. The four squares in each horizontal row correspond to the four sample groups. The light blue boxes indicate different enzymes. The bar graph represents the relative metabolic content.

In plants, a class of compounds called phytoalexins are synthesized and accumulated in response to biological or non-biological stress. Phytoalexins are often compounds in terpenes, phenols and alkaloids. Flavonoids, a large class of phenolic compounds, are derived from a branch of phenylpropanoid biosynthesis [24]. To identify DEGs associated with phytoalexin synthesis in *Ca. oleifera* leaves infected with *Co. gloeosporioides*, the phenylpropanoid and flavonoid biosynthesis pathway were jointly analyzed using their transcription levels from the transcriptome data. Most genes encoding phenylpropanoid and flavonoid biosynthesis enzymes were significantly up-regulated in both the resistant and susceptible lines after infection with *Co. gloeosporioides*. Interestingly, some genes, encoding *cinnamoyl-CoA reductase* (*CCR*), *flavonol synthase* (*FLS*), *flavanone 5-hydroxylase* (*F5H*), *glycosyl transferase* (*GT*) and *shikimate hydroxycinnamoyl transferase* (*HCT*) were expressed at higher levels in the infected resistant leaves than in the infected susceptible leaves (Figure 7A).

In plants, phenylpropanoid metabolism is one of the most important secondary metabolic pathways. Plants have evolved a variety of branches of phenylpropanoid metabolism, producing flavonoids, lignans, cinnamic acids, amides and other metabolites. These secondary metabolites play important roles in plant-pathogen interactions [32,33]. Among them, catechins are a widely distributed class of flavonoids, and the biosynthetic pathways of epicatechin and (+)-catechin are basically clarified [34]. The DEGs and DAMs from our omics datasets were mapped to the KEGG pathways and drawn as a network of genes and metabolites. The phenylpropanoid pathway begins with three key enzymes: phenylalanine ammonia-lyase (PAL), trans-cinnamate 4-monooxygenase (C4H), 4-coumarate-CoA ligase (4CL). In this study, *PAL*, *C4H* and *4CL* genes were all highly expressed after *Co. gloeosporioides* infection (Figure 7B). Naringenin can enter two branches through different enzymes: the phenylalanine metabolic pathway and the flavonoid metabolic pathway. The catalytic product of flavonoid 3-hydroxylase (F3′H) is the direct precursor of flavonols and the intermediate product of anthocyanins and catechins. The *F3′H* gene had the highest transcript levels in infected resistant leaves (Figure 7B). The degree of hydroxylation of the B-ring of flavonoids is closely related to the antioxidant function of the product [35]. Within the flavonoid pathway, dihydroflavonol 4-reductase (DFR) is a key enzyme for the synthesis of anthocyanins, catechins and proanthocyanidins and controls the flux through these three branches [36]. Two *DFR* genes were highly expressed after infection (Figure 7B). Leucoanthocyanidin reductase (LAR) and anthocyanidin synthase (ANS) compete for the same substrate (leucocyanidin). The leucocyanidins are converted into catechins or gallocatechins by LAR or into cyanidins, the precursors of epicatechins and epigallocatechins, by ANS. Our results showed that the *LAR* genes had higher transcript levels than the *ANS* gene. Anthocyanidin reductase (ANR) reduces anthocyanins into the corresponding epicatechins and epigallocatechins. An *ANR* gene (c109855.graph_c0) was obtained and showed the highest level of expression in infected leaves of the resistance and susceptible. Shikimate hydroxycinnamoyl transferase (HCT) catalyzes the transfer of the p-coumaroyl from p-coumaroyl-CoA to shikimate [37]. Two *HCT* genes showed opposite expression trends before and after infection; c110487.graph_c0 increased after infection, while c106589.graph_c0 decreased after infection. Other genes in these related pathways, such as *CCR*, *CADs* and *F5H*, showed consistent trends and were highly expressed after infection (Figure 7B).

### 2.7. qRT-PCR Verification of Gene Expression

**Figure 8:**
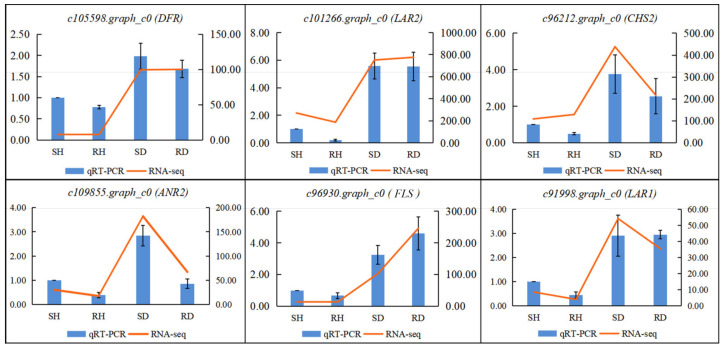
The relative expression levels of six selected DEGs were compared by RNA-seq and qRT-PCR. The line chart shows the gene expression level from the transcriptome (FPKM); The qRT-PCR expression levels were calculated as a ratio relative to the level of expression of uninfected susceptible line CL102 (SH), which was set as 1.

To verify the credibility of the expression levels obtained from the RNA-seq data, qRT-PCR was used to evaluate the transcription levels of six differentially expressed flavonoid-related genes (Figure 8). The expression trends of *DFR* (c105598.graph_c0), *LAR1*(c91998.graph_c0), *LAR2* (c101266.graph_c0), *CHS* (c96212.graph_c0), *ANR* (c109855.graph_c0) and *FLS* (c96930.graph_c0) were highly consistent with the transcriptome data. These results supported the reliability of RNA-seq data.

## 3. Discussion

Anthracnose, a fungal disease caused by Colletotrichum spp., is one of the most pervasive and important diseases of *Ca. oleifera* and affects tea-oil yield and product quality, threatening the development of the tea-oil industry in China [38,39]. Breeding disease-resistant varieties is often the best way to manage plant diseases. However, this requires both understanding the interaction between *Ca. oleifera* and Colletotrichum and digging out disease-resistance genes from *Ca. oleifera*. In this study, healthy leaves and diseased leaves of two *Ca. oleifera* lines showing different resistance to anthracnose were analyzed by both transcriptomics and metabolomics. This combined analysis provides a unique opportunity to understand the candidate genes and metabolites involved in the disease resistance pathway of *Ca. oleifera*.

In this study, a total of 5001 DEGs were identified among the four comparison groups, of which 479 down-regulated DEGs were further characterized. These 479 DEGs were mapped to KEGG pathways. Several KEGG pathways were significantly enriched, the most abundant being “tyrosine metabolism”, followed by “phenylpropanoid biosynthesis” and “flavonoid biosynthesis”. These changes in the transcriptome correlated with the metabolomics data. Tyrosine is an aromatic amino acid that is the precursor of various plant metabolites, which have different physiological functions as electron carriers, antioxidants, attractants and defense compounds [40,41]. In our study, both resistant and susceptible lines of DEGs showed higher expression inoculated than uninoculated, and most DEGs were most highly expressed in the inoculated resistant line. This result suggests that the accumulation of tyrosine metabolites may play a key role in the defense against *Co. gloeosporioides* infestation. One tyrosine-derived metabolite family is the flavonoids, which are known to play roles in plant-pathogen interactions [26,42,43]. DEGs in flavonoid metabolic pathways of resistant and susceptible lines showed higher expression inoculated than uninoculated, which was proven by qRT-PCR too. In the *Ca. oleifera* metabolome, 249 metabolites with known structures were identified, of which 68 were differentially accumulated in our four samples, including some metabolites related to tyrosines, flavonoids, alkaloids and hormone synthesis. Additional disease-related metabolites are discussed below.

During the infection and spread of pathogenic bacteria, plants will mobilize a large number of factors for defense, especially metabolites, such as reactive oxygen species and phytoalexins [44]. These reactions in the plant first require a large supply of energy. Therefore, when pathogenic bacteria invade, energy metabolism in plants will increase significantly [45]. Most of the energy in plants comes from respiration, and the substrate of respiration is glucose [45]. In the inoculated plants 72 h of inoculation, glucose in both the resistant and susceptible lines increased compared with the uninfected leaves. Interestingly, the inoculated resistant line had a glucose content 1.3 times greater than that of the inoculated susceptible line. One reason may be that pathogenic bacteria enter plant cells and consume glucose [46]. Therefore, a less resistant line may show lower glucose levels. The non-reducing disaccharide trehalose was also higher in the resistant line, can protect proteins and cell membranes under abiotic stress and can induce plants to produce H_2_O_2_, thereby activating disease resistance genes [47,48]. When infected by *Co. gloeosporioides*, the resistant lines also showed higher levels of L-tyrosine, 3-hydroxydodecanoic acid, stearic acid and procyanidin B2. The accumulation of defense-related tyrosine in plants subjected to biotic and abiotic stresses has been well documented [49,50,51]. L-tyrosine is the precursor of lignin, which can affect the penetration of bacteria through strengthening the cell wall, affecting the formation of the haustorium within the cell [52,53]. Stearoylcarnitine, which has certain antibacterial properties, was increased. Stearic acid and stearoylcarnitine, as a fatty acid and its derivatives, have been found to be resistance-related compounds that enhance an induced systemic resistance response and prevent the penetration and proliferation of pathogens by strengthening cell walls and membranes [54]. On the other hand, fatty acids and their derivatives are some of the main components of the stratum corneum and wax, which can prevent harmful substances and pathogens from easily entering the host [55]. Procyanidin B2 protects plant cell tissues from peroxidative damage by scavenging free radicals produced by plants when they are subjected to stress. Procyanidins have been shown to have some inhibitory effects on the growth of pathogenic bacteria [56,57,58].

Amino acids are required for protein synthesis, including disease-responsive proteins [59]. The amino acid levels were significantly increased in both susceptible and resistant lines with inoculation. Among the differentially accumulated amino acids, the most changes were argininosuccinic acid, an intermediate of the guanylate cycle which synthesizes arginine and fumaric acid [60,61,62]. Fumaric acid is an intermediate metabolite in the tricarboxylic acid cycle and participates in metabolic reactions, such as sugar and fat metabolism in plant cells [62]. Arginine can be converted into polyamines (PAs) or NO. As signaling factors, polyamines can affect protein synthesis and improve plant disease resistance [63], while NO can stimulate the Ca^2+^ pool to regulate the expression of downstream disease resistance genes, such as increasing antioxidant enzyme activity to reduce oxidative damage [64]. At the same time, arginine, glycine and aspartic acid can be used as signals to connect the cell wall and cell membrane, strengthening the defense against pathogens [65]. Among the amino acids, glycine is the best energy source for *Co. gloeosporioides* [66]. L-threonine, L-leucine, and L-tyrosine can inactivate the toxins released by pathogenic bacteria, reducing their pathogenicity [67].

In the inoculated lines, the fatty acid stearic acid was a differentially accumulated metabolite. Many other precursor substances with antibacterial activity were also produced, such as cyclopentanone derivatives. Stearic acid (octadecanoic acid) can generate linolenic acid [68]. Linolenic acid is the precursor of the plant disease resistance signaling molecule jasmonic acid [68]. At the same time, jasmonic acid can induce plants to produce antibacterial substances, such as tannins, flavonoids, and total phenols and can increase the activity of defensive enzymes [69]. The phenolic compound epicatechin is an inhibitor of pathogenic bacteria and can inhibit the germination of spores and growth of mycelia, showing the highest content in the inoculated resistant line [58,70]. The plant-specific polyphenol rhapontigenin has bactericidal, anti-inflammatory and anti-oxidant properties and was 2.76 times in the resistant line compared to the susceptible line after inoculation. The alkaloids 1S, 9R-hydrastine and oxyquinoline, which have bactericidal and anti-oxidant effects, were 3.85 times and 4.43 times higher in the resistant line the in the susceptible line after inoculation [71]. Kaempferol 3-O-rutinoside, a flavonoid and derivative of quercetin, also has a certain antibacterial effect [72]. This increase in the content of certain metabolites in the disease-resistant clones may increase the plant’s chance to ward off the pathogenic bacteria. Some compounds, like 1S, 9R-Hydrastine, were identified in both the inoculation leaves and uninoculation leaves, indicating that they may be pre-made antibacterial compounds in uninfected plants [71]. 

**Figure 9:**
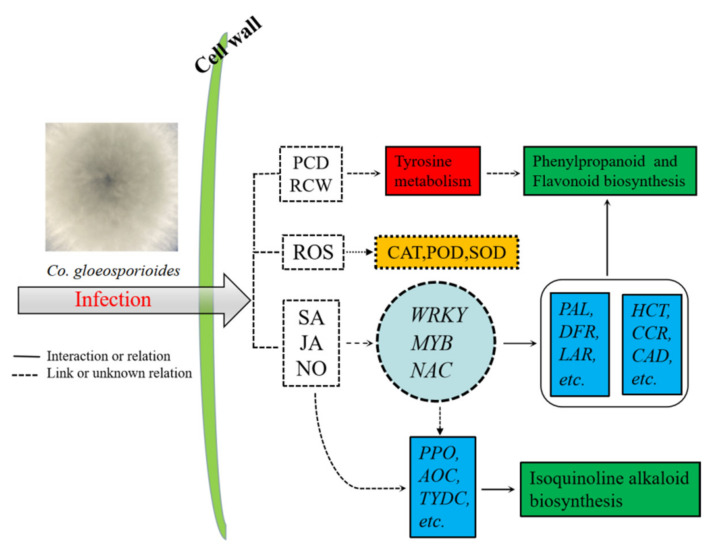
A schematic diagram of the defense response of *Ca. oleifera* to the anthracnose pathogen.

Based on our transcriptomic and metabolomic datasets, the potential defense network within *Ca. oleifera* leaves against *Co. gloeosporioides* infection was delineated (Figure 9). When leaves of *Ca. oleifera* are infected by *Co. gloeosporioides*, metabolic pathways related to tyrosine are activated. These metabolic pathways can lead to excessive accumulation of reactive oxygen species (ROS), which activates the peroxidase system, including enzymes like POD, SOD and CAT. At the same time, these cellular activities lead to the production of signal transduction molecules, such as JA, SA, and NO. Then, downstream secondary and tertiary regulatory networks are activated, mainly through transcription factors (TFs). Finally, these TFs induce the expression of important downstream functional genes that produce disease-resistance metabolites (including flavonoids, alkaloids, phytoalexins, etc.). Our data suggested that flavonoid biosynthesis plays a decisive role in the defense and activation of immunity against anthracnose in *Ca. oleifera*. However, the disease resistance of *Ca. oleifera* is a combination of complex regulation and signaling mechanisms. Further research should focus on how flavonoids and related metabolites, as well as the related regulatory genes, participate in disease resistance in *Ca. oleifera*. Overall, the study explored the defense mechanism of *Ca. oleifera* against anthracnose, which will contribute to mining the molecular markers of anthracnose resistance in this woody plant and to the cultivation of *Ca. oleifera* varieties highly resistant to anthracnose.

## 4. Materials and Methods

### 4.1. Plants, Fungal Materials and Treatments

Two-year-old cuttings of two lines of *Ca. oleifera*, the resistant cultivar CL150 and the susceptible cultivar CL102, were grown in the Climate Chamber of the Research Institute of Subtropical Forestry, Chinese Academy of Forestry, (CAF, N30°05′, E119°96′), Hangzhou, China. The plants, transplanted into plastic basins (14 cm in diameter × 11 cm in height), were maintained at 26 °C and a relative humidity of 90% with cycles of 16 h light and 8 h darkness. The pathogenic *Colletotricum gloeosporioides* Penz., provided by the research group of forest protection of Central South University of Forestry and Technology, was cultured on potato dextrose agar (PDA) for 5–7 days [73]. When the plate was full of mycelium, agar plus bacteria was taken from the edge of the colony with a 5-mm sterilized punch [73].

*Ca. oleifera* young leaves were selected for inoculation with *Co. gloeosporioides*. The inoculation was carried out on the third and fourth new leaf (from top to bottom). The surface of each leaf was sterilized with 75% ethanol and then washed with sterile water. A sterile pin was used to puncture the leaves on both sides of the main vein (two wounds per leaf), a drop of 10 μL of sterile 1% glucose was added to each wound, and then the puncture was covered with a 5-mm disc of fungus mycelium. Each treatment was done on six seedlings per line, for a total of 12 leaves. The puncture wounds on uninoculated plants from the two lines were covered with sterile PDA plugs. 

### 4.2. Phenotypes of Ca. oleifera after Infection by Co. gloeosporioides

The diameters of the disease spots on the leaves were observed and recorded 12 h, 24 h, 48 h, 72 h, 96 h and 120 h after inoculation with the agar plugs plus bacteria. There were twelve biological repeats per treatment (Six leaves, each with two agar plugs). 

### 4.3. Transcriptional Profiling

After 72 h of infection, more than 500 mg of leaves were collected for RNA extraction. Total RNA was isolated using TRIzol reagent (Promega, Beijing, China) according to the manufacturer’s instructions. A total amount of 1 μg RNA per sample was used as input material for generation of the sequencing libraries using NEBNext^®^Ultra™ RNA Library Prep Kit for Illumina^®^ (NEB, Ipswich, MA, USA). In total, 12 RNA-Seq libraries, namely four treatments consisting of uninoculated and inoculated plants of the resistant and susceptible lines (RD, RH, SD and SH) with three replications of each combination, were separately constructed. Library quality was assessed on an Agilent Bioanalyzer 2100 (Agilent, Santa Clara, CA, USA). The library was subjected to 125 bp/150 bp paired-end sequencing by Biomarker Technologies (Peking, China) on an Illumina Hiseq 2000 platform (San Diego, CA, USA). Gene function was annotated based on the following databases: Nr (http://www.ncbi.nlm.nih.gov, accessed on 8 February 2021); COG (http://www.ncbi.nlm.nih.gov/COG/, accessed on 8 February 2021); Swiss-Prot (http://www.uniprot.org/, accessed on 8 February 2021); GO (http://www.geneontology.org/, accessed on 8 February 2021); KEGG (http://www.genome.jp/kegg/, accessed on 8 February 2021) and KOG (http://www.ncbi.nlm.nih.gov/KOG/, accessed on 8 February 2021). All raw sequences used in this study can be found in Sequence Read Achieve (SRA) of the National Center for Biotechnology Information (NCBI) database (BioProject accession: PRJNA775660). TBtools were used to create the HeatMap and Venn plots.

#### 4.3.1. Differentially Expressed Gene Analysis

Gene expression levels were estimated by RSEM for each sample [74]. The Fragments Per Kilobase of transcript per Million mapped reads (FPKM) value was used to indicate the expression abundance of the corresponding Unigene. Four pairwise comparisons were made from the RNA-seq data, namely inoculated vs. uninoculated resistant line CL150 (RD/RH); inoculated vs. uninoculated susceptible line CL102 (SD/SH); inoculated resistant line CL150 vs. inoculated susceptible line CL102 (RD/SD) and uninoculated resistant line CL150 vs. uninoculated susceptible line CL102 (RH/SH). Differential expression analysis of the two conditions/groups was performed using the DESeq R package (1.10.1). FDR < 0.01 and |log2 Fold Change| > 1 were used as the criteria for screening DEGs. Where Fold Change indicates the ratio of expression between two samples (groups). 

#### 4.3.2. Quantitative Real-Time Polymerase Chain Reaction

We selected six differentially expressed flavonoid metabolism-related unigenes for quantitative real-time PCR (qRT-PCR) analysis. The *CoGAPDH* gene was used as an internal reference gene [75]. Specific primers were designed using Premier Express 3.0.1 and are listed in Supplementary Table S4. RNA extraction, cDNA synthesis and real-time quantitative reverse transcription were performed according to Jin et al. [76]. Briefly, the leaves were collected for RNA extraction, RNA samples were used to synthesize cDNA, and a Step One Plus Real Time Fluorescent Quantitative PCR system was used to monitor the amount of cDNA. Assays of each gene were repeated three times. Quantification was evaluated using the 2^−(ΔΔCt)^ method [77].

### 4.4. Metabolomics Analysis

Seventy-two hours after inoculation, the leaves were collected, with six biological repeats per treatment. Each sample (200 mg) was snap-frozen with liquid nitrogen, dried using vacuum desiccation, and crushed using a mixer mill. 50 mg of sample was taken and placed in an EP tube, then add 1000 μL extraction solvent Containing an internal target (V methanol: V acetonitrile: V water = 2:2:1, which was kept at −20 °C before extraction). Homogenized in ball mill for 4 min at 45 Hz, then ultrasound treated for 5 min (incubated in ice water). After homogenization for 3 times, incubation for 1 h at −20 °C to precipitate proteins. Then centrifuged at 12,000 rpm for 15 min at 4 °C transfer the supernatant (500 μL) fresh into EP tubes, dry the extracts in a vacuum concentrator without heating, add 100 μL extraction solvent (V acetonitrile: V water = 1:1) reconstitution. vortex 30 s and sonicate 10 min (4 °C water bath), centrifuge for 15 min at 12,000 rpm, 4 °C transfer the supernatant (60 μL) into a fresh 2 mL LC/MS glass vial, take 10 μL from each sample and pooling as QC samples, take 60 μL supernatant for the UHPLC-QTOF-MS analysis. UHPLC-Q-TOF/MS analyses were performed using an UHPLC system (1290, Agilent Technologies, Santa Clara, CA, USA) with a UPLC BEH Amide C18 column (1.7 μm 2.1 × 100 mm, Waters, Milford, MA, USA) coupled to TripleTOF 6600 (Q-TOF, AB Sciex, Framingham, MA, USA). The mobile phase consisted of 25 mM NH4OAc and 25 mM NH_4_OH in water (pH = 9.75) (A) and acetonitrile (B) was carried with elution gradient as follows: 0 min, 95% B; 7min, 65% B; 9 min, 40% B; 9.1 min, 95% B; 12 min, 95% B, which was delivered at 0.5 mL min^−1^. The injection volume was 2 μL. The Triple TOF mass spectrometer was used for its ability to acquire MS/MS spectra on an information-dependent basis (IDA) during an LC/MS experiment. In this mode, the acquisition software (Analyst TF 1.7, AB Sciex) continuously evaluates the full scan survey MS data as it collects and triggers the acquisition of MS/MS spectra depending on preselected criteria. In each cycle, 12 precursor ions whose intensity greater than 100 were chosen for fragmentation at collision energy (CE) of 30 V (15 MS/MS events with product ion accumulation time of 50 msec each). ESI source conditions were set as follows: Ion source gas 1 as 60 Psi, Ion source gas 2 as 60 Psi, Curtain gas as 35 Psi, source temperature 650 °C, Ion Spray Voltage Floating (ISVF) 5000 V or −4000 V in positive or negative modes, respectively.

MS raw data (.wiff) files were converted to the mzXML format using ProteoWizard which parameters were set to process the 0–12 min Rt range of the chromatograms and m/z domain of mass range 70–1200 Da and processed by R package XCMS (version 3.2) with minfrac set to 0.5 and cutoff set to 0.6. The preprocessing results generated a data matrix that consisted of the retention time (RT), massto-charge ratio (m/z) values, and peak intensity. R package CAMERA was used for peak annotation after XCMS data processing. In-house MS2 database was applied in metabolites identification. The different metabolite list for each comparison was separately submitted into the MBRole (http://csbg.cnb.csic.es/mbrole/, accessed on 8 February 2021), METLIN (http://metlin.scripps.edu/index.php, accessed on 8 February 2021), HMDB (http://www.hmdb.ca/, accessed on 8 February 2021), ChEBI (https://www.ebi.ac.uk/chebi/init.do, accessed on 8 February 2021), and PubChem (https://www.chemeurope.com/en/, accessed on 8 February 2021) databases. Pathway enrichment analysis was based on the KEGG (https://www.kegg.jp/kegg/pathway.html, accessed on 8 February 2021) database. Multi-dimensional statistics (VIP > 1.0) were used, and when the multiple of difference was greater than 2 or less than 0.5, a single-dimensional analysis (*t*-test) was added, with *p* < 0.05 as the screening criterion for naming DAMs.

### 4.5. Combined Metabolomic and Transcriptomic Analysis

Pearson correlation coefficients and corresponding *p*-values were used to screen metabolites and related genes within the combined metabolomic and transcriptomic analysis. The screening criteria were PCC > 0.80 and PCCP < 0.05 [78]. To better understand the relationship between genes and metabolites, we mapped the differentially expressed genes and metabolites among the same treatments (RD, RH, SD and SH) to their associated KEGG pathways.

### 4.6. Statistical Analysis

Phenotypic data were processed in Microsoft Office Excel 2016. Statistical analysis was performed using SPSS (version 22.0). One-way ANOVA followed by Duncan’s multiple tests were used to test the significance of difference at the = 0.05 probability level. Each variety was divided into an inoculated group (RD, SD) and uninoculated group (RH, SH). Transcriptome data analysis was performed using BMKCloud (www.biocloud.net, accessed on 8 February 2021).

## 5. Conclusions

Cultivating disease-resistant varieties of *Ca. oleifera* is the most economical and likely most effective measure to control anthracnose. In this study, we analyzed the CL150 and CL102 lines at the phenotypic, metabolic and transcriptional levels both with and without infection by *Co. gloeosporioides*. The correlation between gene expression and metabolite biosynthesis was explored. The results indicated that flavonoid biosynthesis may play an important role in the fight against anthracnose. While this study provides valuable molecular information about the response of *Ca. oleifera* to *Co. gloeosporioides* infection and will greatly promote the selection of resistant varieties using biomarker-assisted selection, the key regulatory genes of flavonoid biosynthesis their functions in disease resistance still need to be further explored and verified.

## Figures and Tables

**Table 1:** Results of KEGG pathway enrichment analysis.

Pathway Name	Number of Down-Regulated DEGs with Pathway Annotation				
	Pathway ID	RD/RH	SD/SH	RD/SD	RH/SH
Tyrosine metabolism	ko00350	10	10	4	1
Phenylpropanoid biosynthesis	ko00940	20	22	8	2
Flavonoid biosynthesis	ko00941	10	17	1	0
Isoquinoline alkaloid biosynthesis	ko00950	6	6	2	0
Galactose metabolism	ko00052	15	9	7	5
Phenylalanine, tyrosine and tryptophan biosynthesis	ko00400	6	8	1	1
Phenylalanine metabolism	ko00360	6	10	3	0
Tropane, piperidine and pyridine alkaloid biosynthesis	ko00960	4	4	2	0
Glutathione metabolism	ko00480	14	9	3	0
Betalain biosynthesis	ko00965	1	1	0	0
Sesquiterpenoid and triterpenoid biosynthesis	ko00909	3	3	1	1
beta-Alanine metabolism	ko00410	10	6	4	1
Glycine, serine and threonine metabolism	ko00260	7	8	3	0
Limonene and pinene degradation	ko00903	1	1	1	1
Ubiquinone and other terpenoid-quinone biosynthesis	ko00130	3	5	2	1

## Data Availability

Data available in a publicly accessible repository that does not issue DOIs Publicly available datasets were analyzed in this study. This data can be found here: (https://www.ncbi.nlm.nih.gov/bioproject/PRJNA775660/BioProject accession: PRJNA775660, accessed on 8 February 2021).

## Supplementary Materials

The following are available online at https://www.mdpi.com/article/10.3390/ijms23010536/s1.

## Abbreviations

DEG	Differentially expressed gene
DAM	Differentially accumulated metabolite
GO	Gene Ontology
KEGG	Kyoto Encyclopedia of Genes and Genomes
POD	Peroxidase
SOD	Superoxide dismutase
CAT	Catalase
ROS	Reactive oxygen species
SA	Salicylic acid
JA	Jasmonic acid
PCD	Programmed cell death
RCW	Reinforcement of the cell wall
PAL	Phenylalanine ammonia-lyase
C4H	cinnamate 4- hydroxylase
CHS	chalcone synthase
CHI	chalcone isomerase
DFR	dihydroflflavonol 4-reductase
ANS	anthocyanidin synthase
4CL	4-coumarate-CoA ligase
F5H	flavanone 5-hydroxylase
F3′H	flavonoid 3′-hydroxylase
F3′5′H	flavonoid 3′,5′-hydroxylase
LAR	leucoanthocyanidin reductase
ANR	anthocyanidin reductase
HCT	spermidine hydroxycinnamoyl transferase
CCR	cinnamoyl-CoA reductase
CAD	cinnamyl alcohol dehydrogenase
FLS	flavonol synthase
GT	glycosyl transferase
h	hour
